# Publisher Correction: Economic impacts of melting of the Antarctic Ice Sheet

**DOI:** 10.1038/s41467-022-35107-6

**Published:** 2022-11-25

**Authors:** Simon Dietz, Felix Koninx

**Affiliations:** 1 London School of Economics and Political Science (LSE), London, UK; 2 Arup, Bristol, UK

**Keywords:** Environmental economics, Cryospheric science, Climate-change adaptation

Correction to: *Nature Communications* 10.1038/s41467-022-33406-6, published online 03 October 2022

The published, original version of this Article contained an error in the final formatting and labelling of panels in Fig. 2.

The original version duplicated both the panel and title of (e) in panel (f) as “RCP8.5, Antarctica”. The correct version labels panel (f) as “RCP8.5, total”, correct image for panel (f) is as follows:
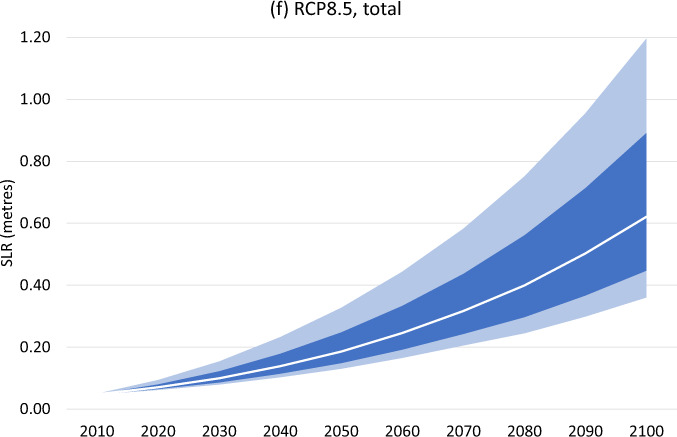


This has been corrected in both the PDF and HTML versions of the Article.

