# Supplementary material for: Economic impacts of melting of the Antarctic Ice Sheet
Source: Nat Commun. 2022 Oct 3;13:5819. doi: 10.1038/s41467-022-33406-6 (PMC9529876; doi:10.1038/s41467-022-33406-6)
Supplement: Supplementary file 1 — Supplementary information [file 41467_2022_33406_MOESM1_ESM.pdf]

Economic impacts of melting of the Antarctic Ice Sheet: Supplementary Information

Simon Dietz[1] and Felix Koninx[2]

[1] London School of Economics and Political Science (LSE), London, UK (corresponding author)

[2] Arup, Bristol, UK

13th September 2022

1. Description of AIS melting model

SMB model

We draw on the work of [1] and [2] to construct a reduced-form model of how increasing global mean surface (GMST) temperatures affect the SMB of Antarctica and the resulting contribution to global mean sea level. Ref. [1] derives a relationship between AIS SMB/accumulation and continental-scale warming using ice-core data, paleo-simulations, and future simulations from the CMIP5 general circulation models and from one high-resolution, regional climate model. The relationship includes an interaction between SMB and dynamic processes. From [2] we obtain the scaling of GMST change to continental-scale temperature change, as well as an adjustment to the predictions of the model of [1] when that model is extrapolated to higher temperatures.

The unadjusted annual mass change ΔSMB at time t is given by

$$\Delta\mathrm{SMB}(t) = \gamma(t - t_0)^{-0.1}\Delta A(t), \tag{1}$$

where t_0 is 2010. $\Delta A(t)$ is the change in continental-scale accumulation from 2010, given by

$$\Delta A(t) = \varphi\omega[\mathrm{GMST}(t) - \mathrm{GMST}(t_0)], \tag{2}$$

where φ is a scaling coefficient of 1.2 that converts GMST change to continental-scale temperature change based on the modelling of [2], and ω is the change in continental accumulation per degree of Antarctic warming, estimated by [1] at approximately 5 +/- 1%/K. We calibrate a normal distribution with a mean of 5% and a standard deviation of 0.4 percentage points. The interaction between SMB and dynamic processes was calibrated by [1] on the response of an ice sheet model to accumulation and can be approximated by $\gamma =$ 7.95mm/yr.

Equations (1) and (2) allow us to estimate the snowfall-induced mass gain for any scenario of global mean temperature change, without needing to rely on runs of a complex ice sheet model. However, [1] only analysed the relationship for continental-scale warming of up to 5K above pre-industrial and temperatures could increase to the extent that SMB in Antarctica turns negative. Ref. [2] reports that the SMB of the ice sheet will turn negative at approx. 7K

warming. To account for this, we model an evolving adjustment factor based on a generalized logistic function:

$$\text{Adjustment}\,(t) = \frac{K - \Delta\text{SMB}(t)}{(C + Qe^{-B(t)})^{1/V}}\,, \tag{3}$$

where *K, C, Q* and *V* are constants, and $B(t) = [GMST(t) - 6.75]$. *K* is calibrated so that the SMB contribution approaches a maximum of 8mm/yr at very high temperatures. This value follows the prognosis from [2] that, above c. 7K warming, Antarctica is committed to losing 70% of its mass via the surface elevation feedback. Seventy per cent of AIS mass is equivalent to c. 40m of SLR and taking a rapid deglaciation of approximately 5,000 years yields a maximum of 8mm/yr SLR.

Combining Equations (1) and (2) with the adjustment factor and cumulating over time yields the adjusted total mass change:

$$\widehat{\text{SMB}}(t) = \sum_{\tau=0}^{t}\left[\Delta\text{SMB}(\tau) + \frac{K - \Delta\text{SMB}(\tau)}{(C + Qe^{-B(\tau)})^{1/V}}\right]. \tag{4}$$

Model of dynamic contributions from basal melting of ice shelves

We model dynamic contributions to SLR from the AIS using the reduced-form model of [3], which is designed to emulate basal ice shelf melting and the resulting contribution of the AIS to SLR in 16 state-of-the-art ice sheet models. The five major ice basins on the continent are modelled separately: East Antarctica, the Ross Sea, the Amundsen Sea, the Weddell Sea, and the Antarctic Peninsula. This is because the dynamic discharge of one basin minimally affects the dynamic discharge of another.

The first step is to translate GMST change relative to pre-industrial *ΔGMST* into subsurface oceanic warming at the mean depth of the ice shelf base in each of the five basins:

$$\Delta T_O(r,t) = \beta(r)\Delta\text{GMST}\big(t - \delta(r)\big). \tag{5}$$

Ref. [3] derived the scaling coefficients *β(r)* and time-delays *δ(r)* from 19 CMIP5 models. Each region of Antarctica thus has 19 possible pairs of scaling coefficients and time delays, drawn at random with equal probability. If *t*=2050 and *δ(r)*=30 years, for example, then the input to Equation (5) is *ΔGMST* in 2020.

The second step is to map subsurface ocean warming into enhanced basal ice shelf melting:

$$\Delta M(r,t) = \lambda \Delta T_O(r,t), \tag{6}$$

where the basal melt sensitivity parameter λ is randomly chosen from a uniform distribution with lower and upper bounds of 7 $\text{ma}^{-1}\text{K}^{-1}$ and 16 $\text{ma}^{-1}\text{K}^{-1}$ respectively. This interval corresponds to values from experimental observations.

The third step translates the enhanced basal ice shelf melting into ice loss/SLR. This utilises reduced-form response functions, which [3] estimated on the behaviour of the 16 ice sheet models. Each ice sheet model was initially subjected to a control run from 1900 to 2100. In this control run, the models were forced with historically observed basal ice shelf melting until 2010 and constant forcing thereafter. After the control run, each ice sheet model was then subjected to an artificial external forcing experiment involving an additional stepwise increase

of 8 m/yr of basal ice shelf melting. The difference in the dynamic contribution to SLR between the experiment and the control run forms the basis of the response function for the particular model and region. The approach assumes that increasing the magnitude of the forcing by a specific factor will increase the magnitude of the response of the ice sheet by the same factor. However, the temporal evolution of the response is not a linear function of time.[a] Response functions can capture the irregular oscillations of ice sheet dynamics in response to external forcing, as illustrated by Figure SI1, which looks at one model/region response function. One must also assume that over the forcing period the five regions of Antarctica respond independently. Ref. [3] showed this is a good assumption. Ref. [3] also subjected the 16 ice sheet models to forcing experiments of 4 m/yr and 16 m/yr of additional basal melting and compared these responses to the main 8m/yr experiment. Generally, there was good agreement between the responses to the step increases of different size.

SLR from dynamic processes S is given by

$$S(r,t) = \sum_{r=1}^{5}\sum_{\tau=0}^{t} \Delta M(r,\tau)R(r,\tau), \tag{7}$$

where R is the value of the response function at time τ, drawn at random from the set of 16 models. The total Antarctic SLR contribution is the sum of Equation (4) and Equation (7).

Ref. [3] derived response functions for the period 1900 to 2100, including providing a hindcast test for the period 1992-2017. They note that "[w]hile individual models may deviate strongly from the observed range, the combination of all models shows a similar contribution for the time period 1992–2017 as was observed with a bias towards slightly higher ice loss" (p46).

The period to 2100 is long enough for many of our purposes in this paper, but not for estimating the social cost of carbon – a large portion of the current social cost of carbon stems from damages after 2100. Therefore, we developed a method of extrapolating the response functions to 2200 using time series analysis techniques. This makes tractable the extrapolation problem in the absence of being able to run the ice sheet models themselves. We treat the dynamic contribution to SLR estimated by each ice sheet model over the period 1900 to 2100 as a time series (e.g., Figure SI1). This is first detrended to achieve stationarity and then the properties of the series are estimated using a moving average function of the first or second order, or an ARMA function of the first or second order, with the model being chosen based on best fit under the Akaike Information Criterion.

[a] Ref. [18] provide more evidence on the applicability of linear response theory to the evolution of ice sheets over the next 200 years. They subjected 10 ice sheet models, characterising both Greenland and Antarctica, to experiments where they changed multiple forcings such as the SMB, basal sliding parameters, and basal ice shelf melting. They found that ice sheet response is strongly linearly dependent on the strength of the forcing. This suggests that internal feedbacks are modest. Furthermore, they found that in experiments with a combined forcing, such as increasing basal ice shelf melting together with increasing SMB, resulted in a very similar response to the sum of responses when only one forcing was considered, and then the other.

Figure SI1. Example response function.

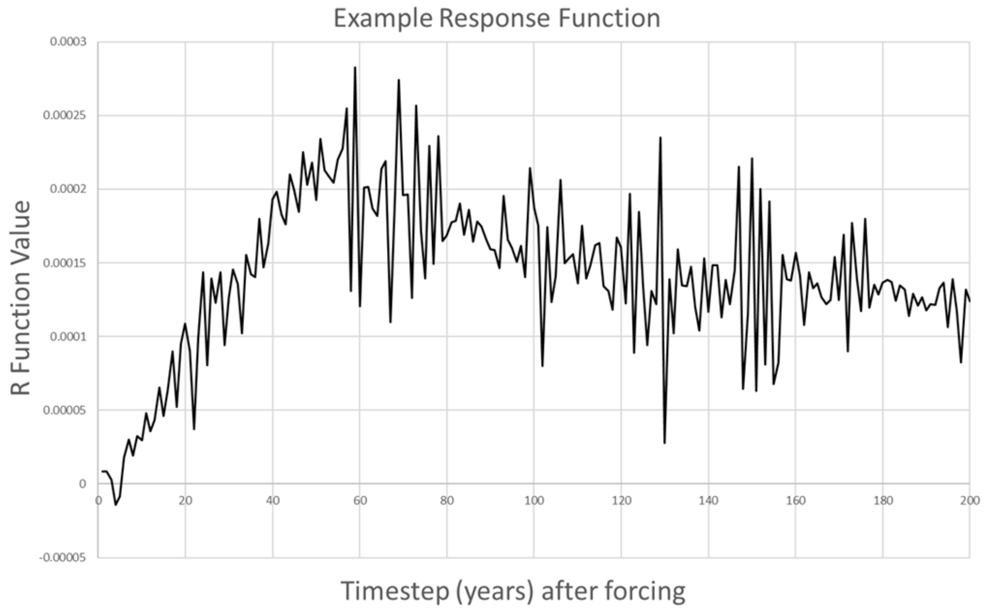

Summary information on uncertain parameters

Figure SI2 provides a pairs plot of the two, continent-wide uncertain scalar parameters, namely the change in continental accumulation per degree of Antarctic warming ω from Equation (2) and the basal melt sensitivity parameter λ from Equation (6). Note the uncertainty is assumed to be uncorrelated. Figure SI3 plots the probability distributions of and pairwise correlations between the CMIP5 scaling coefficients, for each of the five Antarctic regions. Figure SI4 does the same for the CMIP5 time delays. Lastly, Figure SI5 plots the probability distributions of and pairwise correlations between the response functions, for each of the five Antarctic regions. Since the response function is a time vector of uncertain values, we simplify by computing the cumulative SLR response in 2100 to fixed basal ice shelf melting on the RCP4.5 scenario.

Figure SI2. Pairs plot of parameter uncertainty for the change in continental accumulation per degree of Antarctic warming and the basal melt sensitivity.

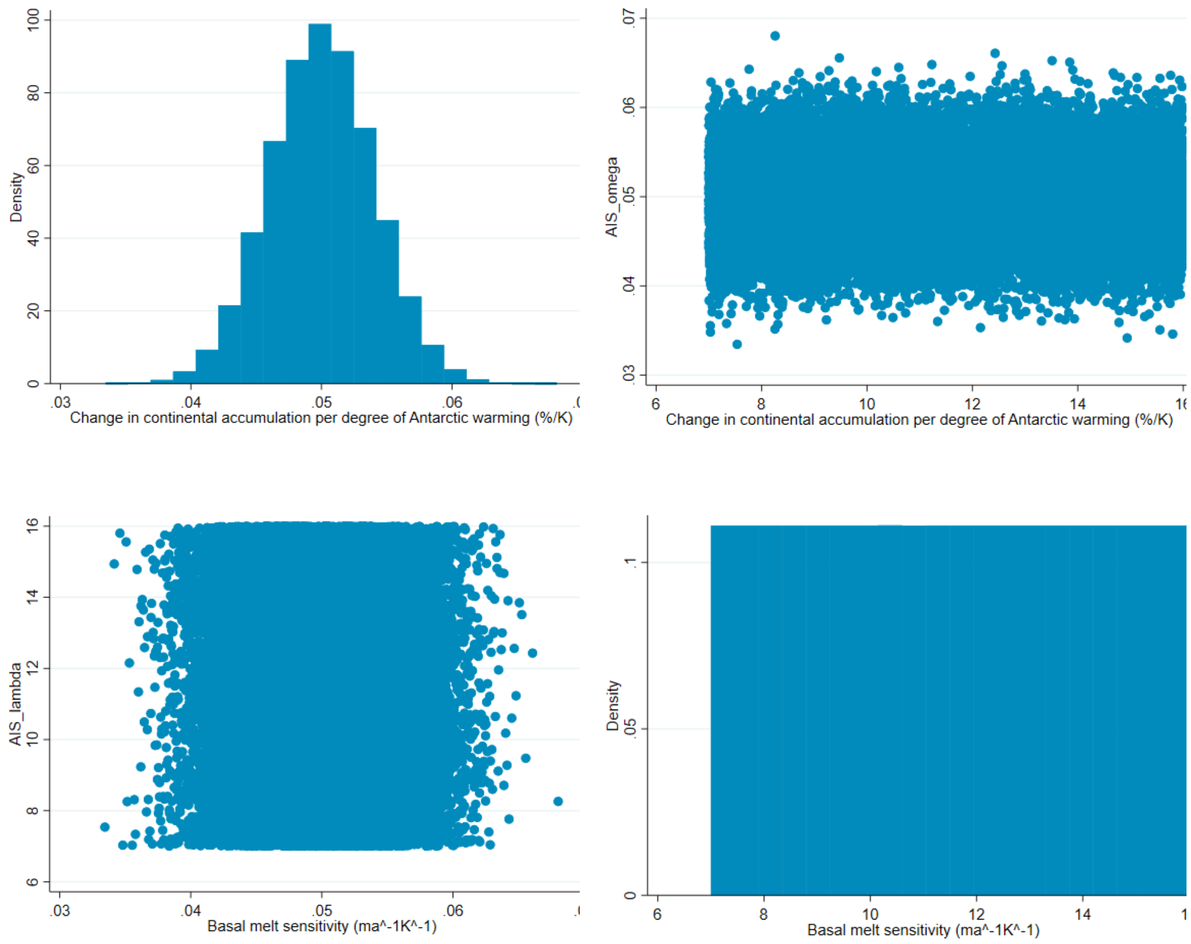

Figure SI3. Probability distributions of and pairwise correlations between the CMIP5 scaling coefficients, for each of the five Antarctic regions.

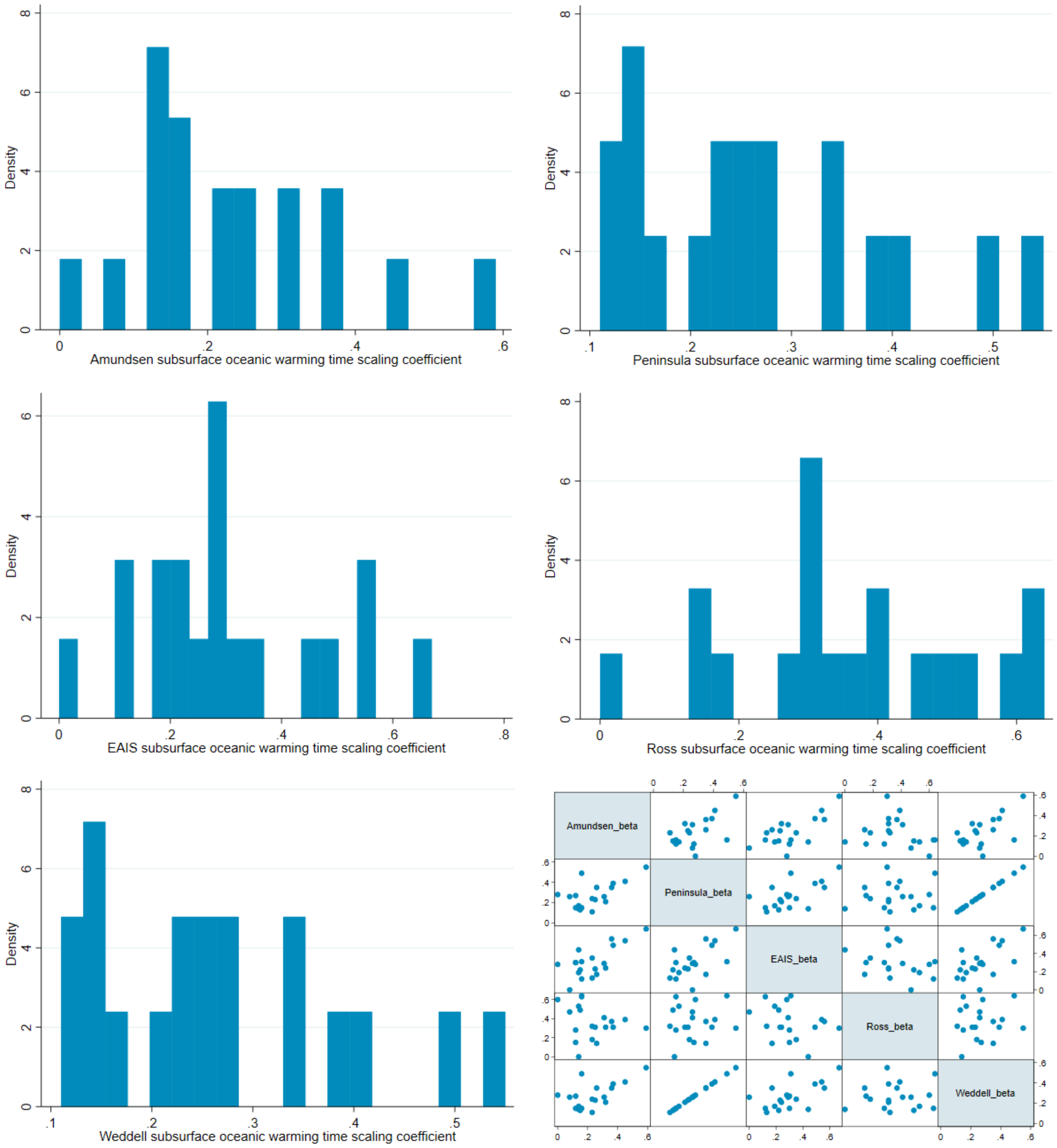

Figure SI4. Probability distributions of and pairwise correlations between the CMIP5 time delays, for each of the five Antarctic regions.

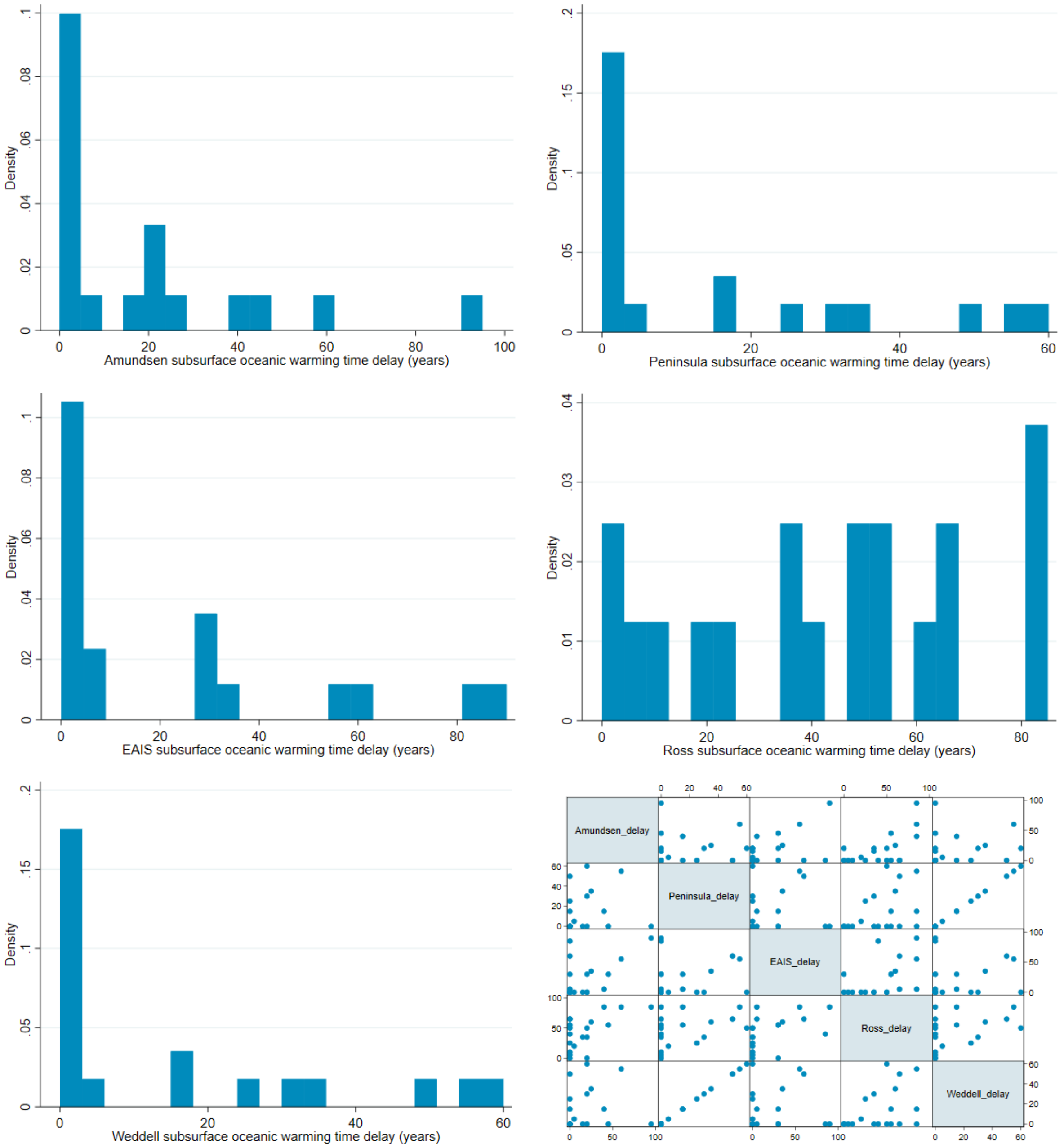

Figure SI5. Probability distributions of and pairwise correlations between the response functions, for each of the five Antarctic regions, computed as the cumulative SLR response in 2100 to deterministic basal melting on RCP4.5, using mean values of all uncertain parameters used in calculating basal melting.

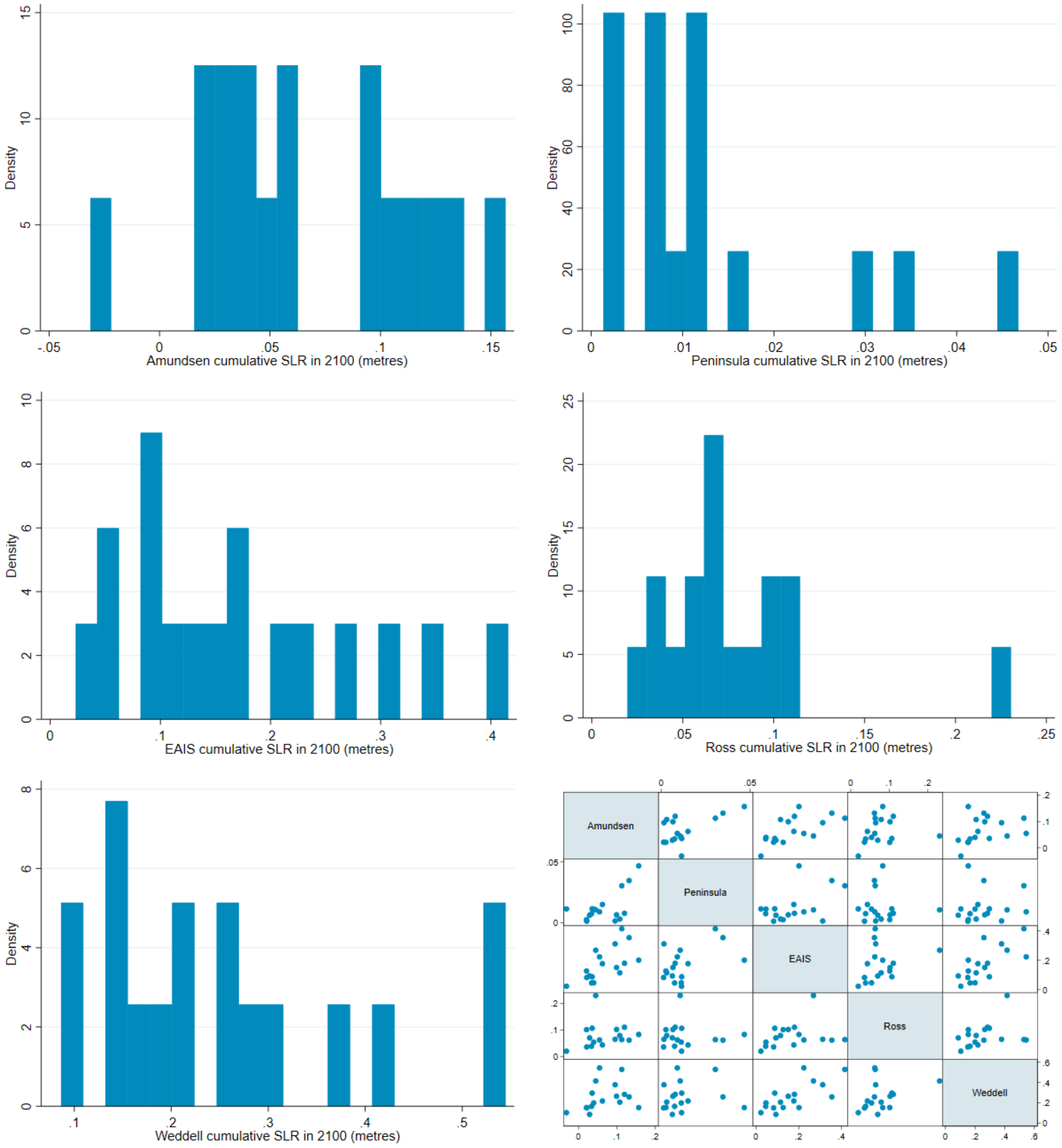

2. Statistical downscaling to local SLR

Global mean SLR from the AIS melting model (and where appropriate from other sources of SLR via the META model) is converted into local SLR at the level of DIVA segments (N=12,148) using a statistical downscaling procedure.

From [4] we obtain a set of datapoints on the x-y plane, where x is global mean SLR and y is SLR for the local segment. The data are decadal median estimates from 2010 to 2200 and pooled across the RCP2.6, 4.5 and 8.5 scenarios, so for each local segment N=60 datapoints. At the segment level, we then regress, using ordinary least squares, the absolute difference in SLR between the segment and the global mean on global mean SLR using a flexible cubic functional form that can capture non-linear and non-monotonic relationships:

$$\begin{aligned}\mathrm{LocalSLR}(i) - \mathrm{GlobalSLR} &= \alpha(i) + \beta_1(i)\mathrm{GlobalSLR} + \beta_2(i)\mathrm{GlobalSLR}^2 \\ &+ \beta_3(i)\mathrm{GlobalSLR}^3,\end{aligned} \tag{8}$$

where *i* denotes the segment. This yields 12,148 sets of estimated coefficients, which are then used to project local SLR in our scenarios.

Table SI1 evaluates the performance of this procedure by reporting goodness of fit to the local SLR projections of [4] on which the functions are estimated. The mean absolute error across segments over the period 2010-2100 is 0.005m on RCP4.5, 0.003m on RCP2.6 and 0.010m on RCP8.5. That is, on average between 2010 and 2100 in any given segment, the downscaled estimate is within 0.005m of the actual estimate on RCP4.5. The corresponding median absolute errors are 0.003m, 0.002m and 0.005m, respectively. Thus, goodness of fit is very good in general, with a tail of segments for which the fit is less good. We also considered an alternative formulation using the ratio of local to global SRL on the left-hand side of the regression equation instead of the absolute difference, but this predictably resulted in a poorer fit at higher levels of absolute global mean SLR.

Table SI1. Goodness of fit of local SLR functions. Statistics are computed over the period 2010-2100 and over all segments without weighting.

	**Scenario**		
**Error**	**RCP2.6**	**RCP4.5**	**RCP8.5**
**Mean absolute (m)**	0.003	0.005	0.010
**Median absolute (m)**	0.002	0.003	0.005
**2.5% absolute (m)**	0.001	0.001	0.001
**97.5% absolute (m)**	0.013	0.018	0.038
**Root mean square**	0.006	0.008	0.017

3. The Coastal Impact and Adaptation Model (CIAM)

CIAM has been built to study the impacts of sea level rise on coastlines worldwide, under different assumptions about adaptation that include the possibility of optimal (least-cost) adaptation planning. The model was introduced and is described in detail in [5].

CIAM works at a high spatial resolution by building on the DIVA database [6]. DIVA partitions the world's coastlines into 12,148 segments with homogeneous physical characteristics. The median length of a segment is 18km. Cost estimates at the segment level can be aggregated to coarser spatial scales, for example the country and global levels. CIAM runs in time steps of 10 years. It is initialised in the year 2000 and can run up to 2200, though we mainly focus on the period up to 2100. The base year currency is 2010 US dollars. We convert this to 2020 US dollars using a decadal inflation rate of 20%.

Each segment in CIAM is described by its physical attributes (coastline length, surface area by elevation, storm surge frequency, wetland extent), socio-economic attributes (income, population density, capital stock), as well as protection costs. Physical attributes are taken from DIVA, while socio-economic attributes and protection costs are derived from a range of sources described in [5]. While some segment attributes such as length are time-invariant, most are time-varying. Future segment population, for example, is based on the United Nations' projections, while future segment income is based on IMF projections.

There are five categories of coastal impact in CIAM:

1. Protection costs (construction of sea walls, dikes, etc.);
2. Retreat costs (relocating people and mobile capital inland, and demolishing immobile capital);
3. Inundation costs (the value of lost land, and immobile capital abandoned);
4. Wetland costs (the value of lost wetland ecosystem services);
5. Flood costs (expected damage to capital stocks and expected mortality from storm surges).

The overall cost of sea level rise is the sum of each of these five cost categories, the first two of which can be regarded as adaptation costs (retreat can further be either proactive or reactive), while the latter three can be regarded as residual damages.

Both overall costs and their composition depends on what is assumed about adaptation. In general, each segment possesses a social planner who solves the following optimisation problem for his/her own segment in each adaptation planning period:

$$\begin{aligned}\mathrm{OverallCost}(i) &= \min_s \sum_{t\in\Delta t}\Bigg[\frac{1}{(1+r)^t}\big(\mathrm{ProtectionCost}(i,s,t)+\mathrm{RetreatCost}(i,s,t)\\ &+\mathrm{InundationCost}(i,s,t)+\mathrm{WetlandCost}(i,s,t)+\mathrm{FloodCost}(i,s,t)\big)\Bigg]\end{aligned}$$

where *s* is the adaptation strategy, *Δt* is the adaptation planning period comprising decadal time-steps *t*, and *r* is the discount rate.

When the adaptation strategy *s* corresponds to no adaptation, *ProtectionCost(t)* = 0 for all *t* and *RetreatCost(t)* is optimally chosen to react to sea level rise at the same time step. Alternatively, when *s* corresponds to optimal adaptation, both *ProtectionCost* and *RetreatCost* are chosen once during each adaptation planning period in order to minimise the discounted *OverallCost* of sea level rise in the current and next adaptation planning periods.

Protection costs are assumed to be linear in the length of the segment's coastline but quadratic in the required height of the defences. Construction costs vary by country to reflect estimated labour and material costs. Protection costs also include the opportunity cost of the land used for protection measures, and operating/maintenance costs. Protection is assumed to be 100% effective up to its designed height, and disamenities such as reduced shore access are ignored.

Retreat literally involves moving inland in the segment. Retreat costs comprise the costs of moving capital and population. These costs are increasing in the amount of capital and population to be moved, respectively, and are also proportional to the distance retreated. Population costs are calibrated based on estimates of domestic migration costs. Capital costs reflect assumptions about how much capital is mobile versus immobile. Mobile capital can be moved at a cost, while immobile capital must be demolished. Retreat takes place fully within a planning period and, once moved, capital is assumed to be just as productive as it was nearer the coast. Reactive retreat is assumed to be five times as expensive as proactive retreat, and accompanying inundation costs are also lower under proactive retreat, because capital is given time to depreciate.

The segment planner's problem is solved independently for each of CIAM's 12,148 segments and sequentially for each adaptation planning period. Thus, when adapting optimally the planner looks forward one period in each planning period. Perfect foresight is assumed, which is unrealistic but necessary for computational tractability given so many optimisation problems are being solved simultaneously. The default adaptation planning periods are 40 years, with shorter initial and final periods, i.e.: 2000; 2010-2040; 2050-2090; 2100-2140; 2150-2180; 2190-2200.

4. Model of Economic Tipping point Analysis (META)

META is a modular IAM that was built to accommodate and evaluate a wide range of climate tipping points. For this study, all climate tipping points included in the original study of [7] are turned off, except for the module simulating melting of the Greenland Ice Sheet, which is essential for evaluating the incremental contribution of the AIS to SLR.

Key features of META relevant to this study are as follows:

Anthropogenic greenhouse gas emissions, growth and population projections

Greenhouse gas emissions and corresponding baseline projections of GDP and population growth are exogenous and taken from the RCP-SSP database. RCP3-PD/2.6 is matched with SSP1, RCP4.5 with SSP2 and RCP8.5 with SSP5. Emissions scenarios are extended beyond 2100 using the Extended Concentration Pathways (ECP) database for emissions [8] and a method was developed of extrapolating the corresponding SSPs beyond 2100. CO2 and CH4 emissions are

modelled explicitly. Other greenhouse gases and forcing agents are combined into an exogenous vector of residual radiative forcing.

Atmospheric chemistry and warming

The FAIR model [9] is used to represent the carbon cycle. Radiative forcing from CO2 is a log function of the contemporaneous ratio of the atmospheric CO2 concentration and the pre-industrial concentration. Radiative forcing from CH4 is modelled explicitly: atmospheric CH4 decays exponentially, while radiative forcing is a square root function of the atmospheric concentration of CH4 in excess of pre-industrial, with co-dependence on atmospheric N2O in the initial model year (2010).

Warming is simulated using a two-box model of heat transfer between the atmosphere and upper oceans, and the deep oceans, which is calibrated on the CMIP5 ensemble [10]. The inputs are radiative forcing from CO2, CH4 and the vector of other GHGs and forcing agents.

Country-level temperature damages

Changes in GMST are disaggregated to the national level using non-linear statistical downscaling. Changes in national mean surface temperature are then fed into non-linear, country-specific damage functions calibrated on recent empirical evidence [11].

SLR from other sources

SLR in META is the sum of contributions from three sources: (1) thermal expansion and melt from glaciers and small ice caps, which is specified as a linear function of warming and uses the calibration of [12]; (2) melting of the Greenland Ice Sheet, replicating the reduced-form model of [13], and; (3) melting of the AIS, using the models described in this paper.

Country-level damages from SLR

To calculate the social cost of carbon from AIS melting in META, CIAM needs to be simplified into something that is computationally tractable within the much larger META model super-structure. CIAM is too complex in two respects: (i) it has too much spatial detail; (ii) it involves endogenous protection/retreat choices at the local level.

Instead, for each country an SLR damage function is specified, which maps SLR into overall economic costs (i.e., the sum of protection, retreat, flood, inundation, and wetland costs),

$$D(j,t) = \theta(j)\mathrm{GlobalSLR}(t), \quad (9)$$

where *D* represents overall costs and $\theta(j)$ parameterises the cost to country *j* per metre of global mean SLR.

To calibrate the country cost coefficients, the full CIAM model is run and aggregated over segments within each country to obtain cost estimates (as a percentage of national GDP) in the (i) no adaptation and (ii) least-cost adaptation scenarios. Costs/SLR in 2050 are used to calibrate the coefficients, based on RCP4.5 and SLR from all sources. A symmetrical triangular distribution is specified for each $\theta(j)$ with a maximum corresponding to costs in scenario (i) and a minimum corresponding to costs in scenario (ii). Thus, adaptation is the source of the uncertainty about each country's cost coefficient, and by using scenarios (i) and (ii) to bound

the distributions, most probability mass is assigned to intermediate scenarios whereby adaptation takes place, but it is sub-optimal. Country distributions are assumed uncorrelated. The functional form of $D(j,t)$ assumes damages are linear in SLR, which is a reasonable approximation of the data in Figures 2 and 3 of the main paper respectively, which show that both SLR and costs of SLR increase more than proportionally with time. Note that the country cost coefficients derive from the CIAM socio-economic scenario, whereas META is based on the SSPs. CIAM's socio-economic scenario has country growth rates close to SSP1 and SSP2 on average, but generally lower than SSP5.

Levels versus growth damages

META adopts a flexible specification allowing damages from temperature and SLR (and in India from the summer monsoon) to affect either the short-term level of GDP, or long-term growth prospects. In the model's main specification, weights of 0.5 are assigned to both damage channels based on the principle of insufficient reason, which accounts for the fact that the empirical evidence on damage channels is only tentative [14].

Consumption and welfare

National GDP per capita is converted into national consumption per capita using country-specific exogenous savings rates, estimated using World Bank data on savings over the period 2005-2015. An isoelastic utility function is specified with a default elasticity of marginal utility of consumption of 1.5, and a utilitarian social welfare functional with a default constant pure rate of time preference of 1%.

6. Supplementary results

Hindcast test

Table SI2 compares the results of hindcasting the model with observations of SLR. Observational data are taken from the IPCC *Sixth Assessment Report* [15] and cover the period 1993 to 2018, the satellite altimetry era. The model is run with observed GMST until 2018. Observed GMST is calculated as the average of the HadCRUT4, ERA5, GISSTEMP and NOAA series. The AIS melting model is initialised in 1900 for all our experiments. Contributions to SLR from other sources (from META) are re-initialised to 1993.

Consistent with [3], we find that the AIS melting model estimates higher SLR between 1993 and 2018 than the observations, although the model's 90% confidence interval is wide and envelopes the corresponding uncertainty about the observations. Conversely, estimated total SLR is lower than the observations, implying that the contributions from thermal expansion, glaciers and small ice sheets, and melting of the Greenland Ice Sheet in META are underestimated relative to the observations. Again, the 90% confidence intervals overlap.

Table SI2. Estimates of SLR from 1993 to 2018 obtained from hindcasting the model (Monte Carlo simulation with sample size 50,000) versus observations obtained from IPCC AR6. Results are the difference in mm from 1993 and both the median and the 90% C.I. are reported.

	**Model estimate**	**Observations**
**Antarctica**	15.9 [3.8 to 51.1]	6.1 [4.0 to 8.3]
**Total (sum all sources)**	41.5 [27.9 to 77.2]	71.2 [60.2 to 82.3]

Components of SLR from Antarctica

Figure SI6 disaggregates the AIS contribution to SLR on the RCP4.5 scenario into the contribution from SMB and the dynamic contributions from each of Antarctica's five regions. SMB reduces SLR by 0.03m in 2100, within a 90% confidence interval of 0.01-0.05m. The largest dynamic contributions are from the Weddell, East Antarctica and Ross regions, in descending order. The positive dynamic contributions far outweigh the negative contribution from SMB.

Figure SI6. Projections of Antarctica's contribution to SLR from different sources on the RCP4.5 scenario, including surface mass balance (top left), and dynamic contributions from each of Antarctica's five regions (top right to bottom right). White line represents median value; dark shaded area represents the 67% confidence internal; light shaded area represents the 90% confidence interval. Results from a Monte Carlo simulation with sample size 50,000.

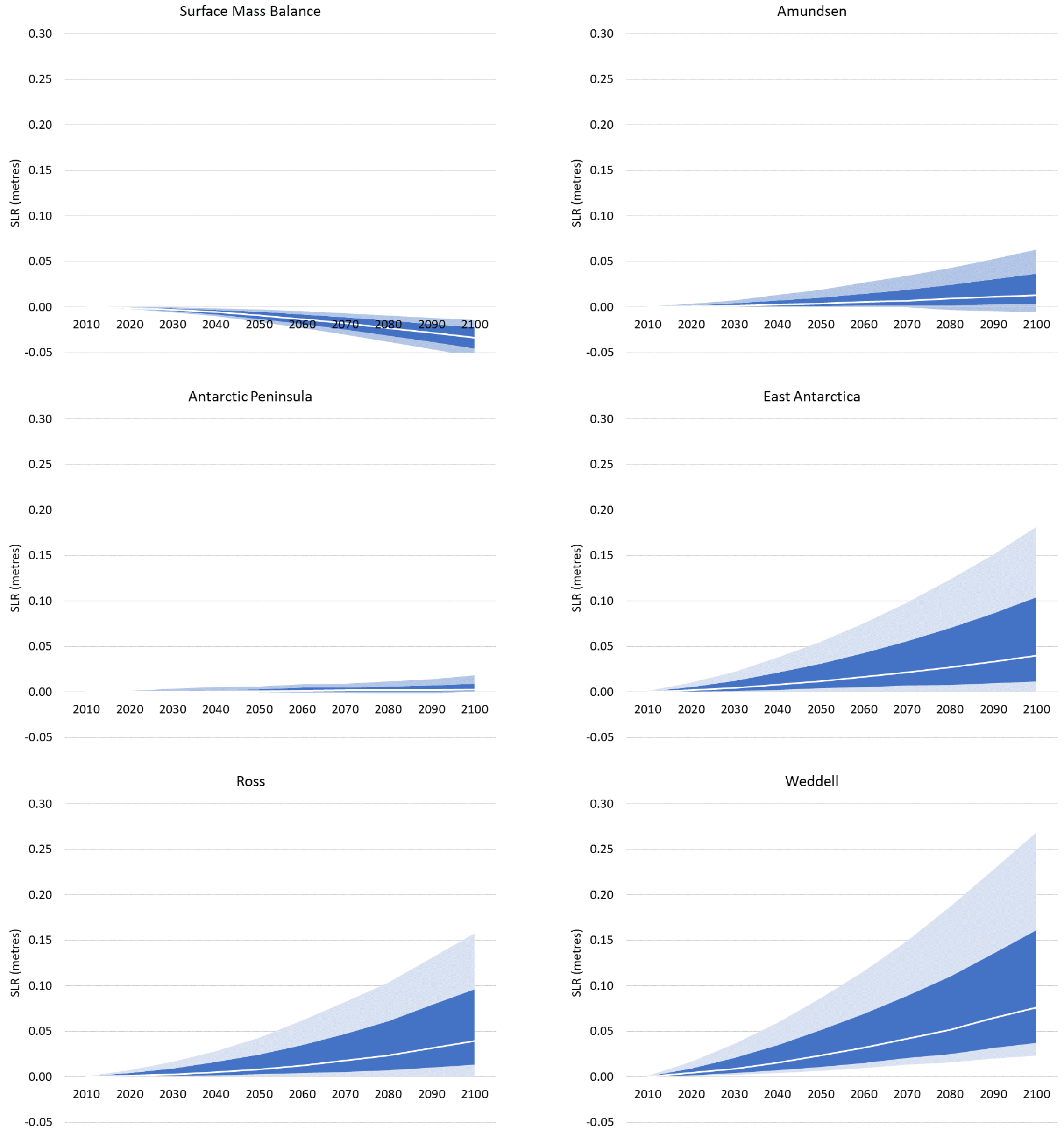

SLR projections: probability and cumulative density functions, and survival functions

Figure SI7 provides more information about the distribution of SLR from Antarctica and from all sources by plotting probability density functions and cumulative density functions for the year 2100. Note the differing scale of the x-axis. Figure SI8 plots survival functions for SLR from Antarctica, where survival is taken to be a threshold value of SLR. We use thresholds of 0.1, 0.2 and 0.3m.

Figure SI7. Probability and cumulative density functions for Antarctica's SLR contribution (left) and total SLR from all sources (right) in 2100 on RCP2.6 (top row), RCP4.5 (middle row) and RCP8.5 (bottom row). Projections are relative to 2000. PDF bins in blue, CDF in red. Results from a Monte Carlo simulation with sample size 50,000.

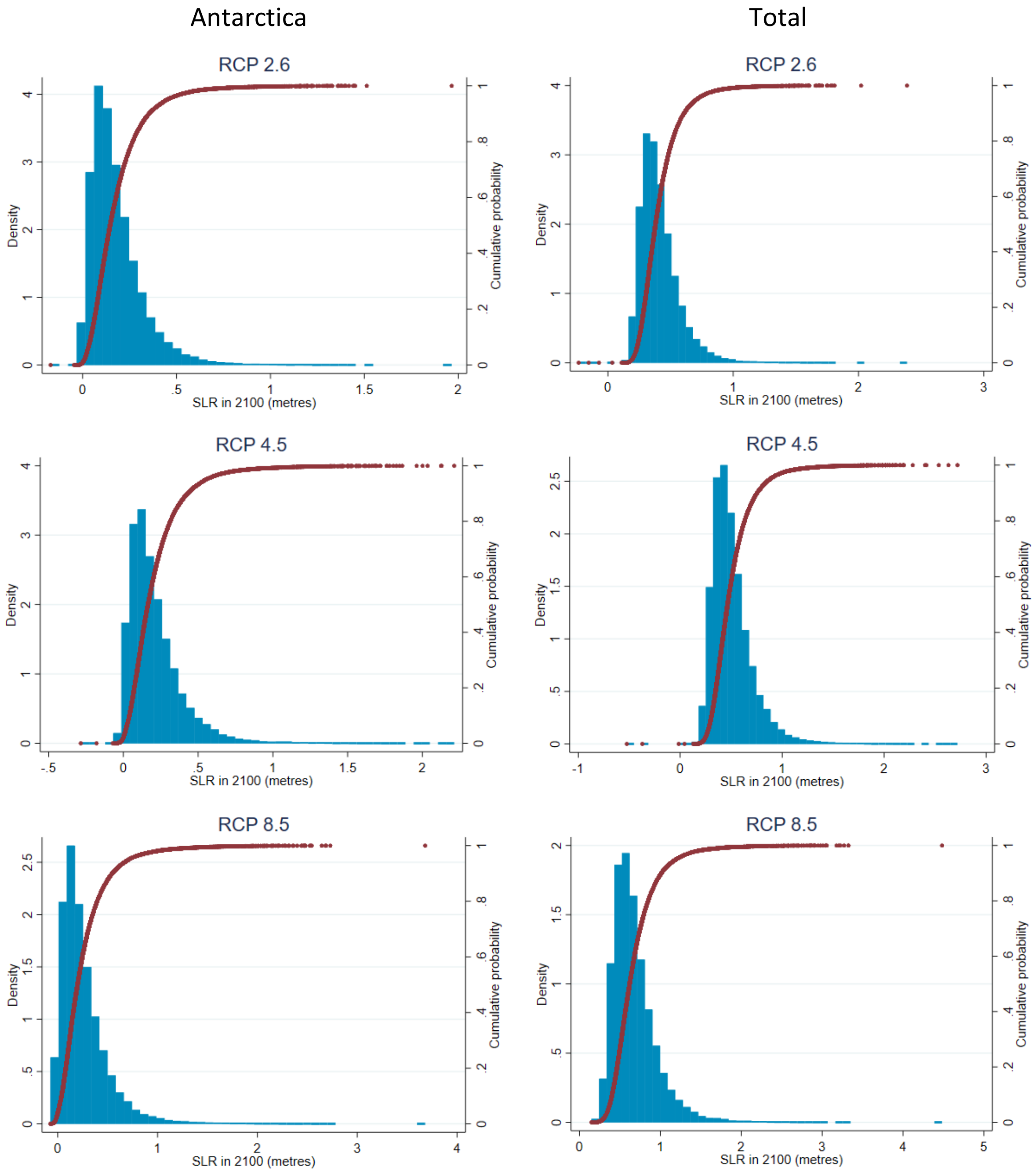

Figure SI8. Survival functions showing the probability that SLR from AIS melting stays below threshold values of 0.1m, 0.2m and 0.3m between 2010 and 2100. Results from a Monte Carlo simulation with sample size 50,000.

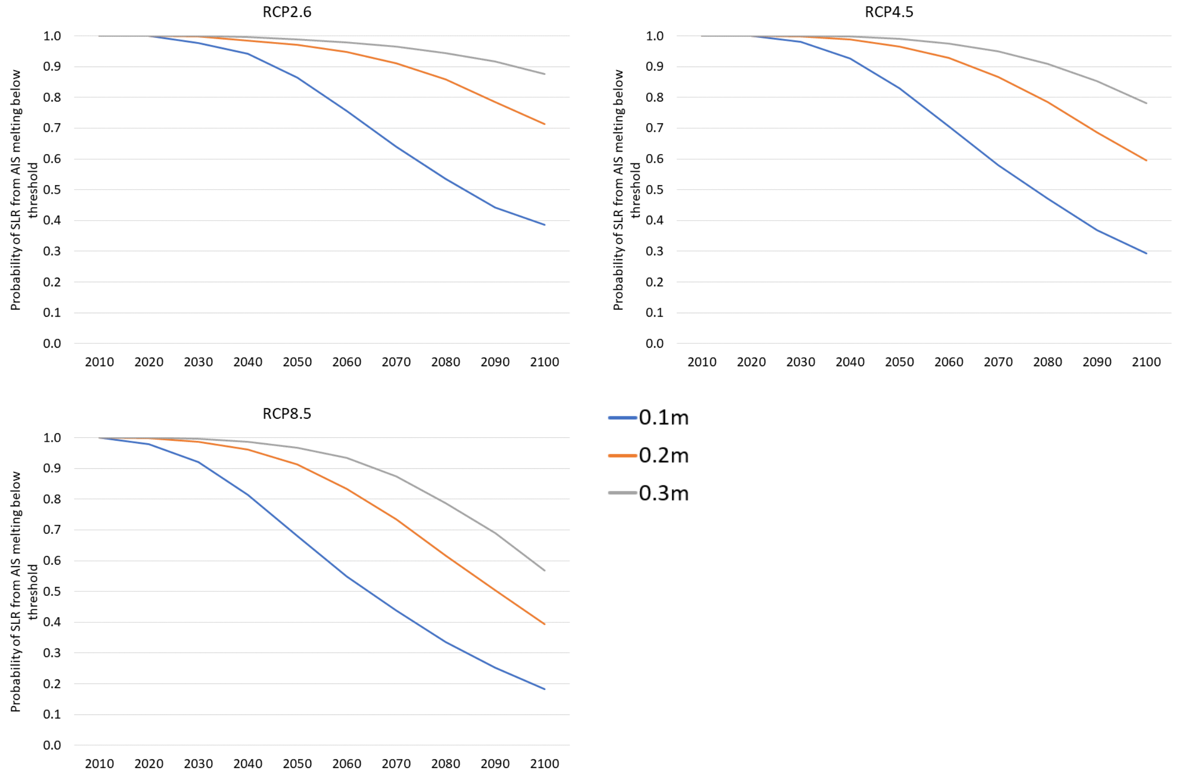

Analysis of Monte Carlo sample size

Table SI3 analyses the consistency of our projections of SLR from Antarctica in 2100 over 5 x 50,000 samples. For this analysis, we use RCP8.5 emissions, as this will provide the stiffest test of numerical convergence. Results agree to within around 0.01m or better, depending on the percentile.

Table SI3. Analysis of consistency of SLR projections over different Monte Carlo samples, focusing on a range of percentiles of the distribution of SLR from Antarctica in 2100. Each sample is of size 50,000 and is generated using a different seed. The emissions scenario is RCP8.5.

	**SLR from Antarctica in 2100**				
**Sample number**	**5%**	**16.7%**	**Median**	**83.3%**	**95%**
**1**	0.008	0.067	0.198	0.434	0.712
**2**	0.008	0.065	0.198	0.437	0.727
**3**	0.007	0.065	0.199	0.436	0.728
**4**	0.008	0.066	0.198	0.432	0.719
**5**	0.007	0.066	0.198	0.437	0.712

Coastal impacts: further analysis

Table SI4 presents the ratio of (undiscounted) costs from AIS melting at the end of the century over those same costs at mid-century, and it presents the same ratios for SLR from other sources. To overcome the aforementioned lumpiness in some of the cost trajectories, the end of the century is taken as 2090 to 2110, while mid-century is 2040 to 2060. On RCP4.5, total costs of AIS melting are 5.2 times higher during the period 2090-2110 than 2040-2060, compared with 4.5x higher for SLR from other sources. On RCP2.6, the corresponding ratios are 5.3x and 4.0x respectively. These results derive from the later contribution of AIS melting to SLR relative to other sources. The exception is RCP8.5, on which the ratios are 4.4x for the AIS contribution and 5.3x for SLR from other sources, which is at least partly attribute to a smaller relative contribution to median SLR from AIS melting.

Table SI4. The ratio of total costs at the end of the century to costs mid-century from AIS melting, compared with other sources. The scenario is no protection. End-of-century costs are averaged over 2090-2110; mid-century costs are averaged over 2040-2060.

	**AIS melting**	**Other sources**
**RCP2.6**	5.25	3.96
**RCP4.5**	5.23	4.50
**RCP8.5**	4.38	5.30

SLR projections from DeConto et al. and ABUMIP

Figure SI9 shows the SLR projections of DeConto et al. [16], who incorporate hydrofracturing and MICI processes into a hybrid ice sheet-shelf model, and the ABUMIP project [17], which forced 15 ice sheet models with an extremely high melt rate underneath the ice shelves.

We reproduce the projections of [16] using the source data behind their Figure 1, which reports SLR as a function of time for each of 109 separate parameterisations. We assign each parameterisation equal probability and construct a distribution by randomly drawing from the set of parameterisations 50,000 times. For the ABUMIP projections [17], we first record the model-specific SLR projections as a function of time contained in their Figure 1(d). They interpret time zero as present-day conditions, so we associate time zero with 2020 (thus, there is zero contribution from AIS melting up to and including 2020). We assign equal probability weights to each model producing an SLR projection in the ABUMIP dataset for the ABUM (extreme sub-ice-shelf melt) scenario. We then randomly draw from the set of models 50,000 times to construct a probability distribution.

The DeConto et al. projections are of median (mean) SLR of 0.34m in 2100 (0.36m), within a 90% C.I. of 0.11-0.63m. The ABUMIP projections are of median (mean) SLR of 1.57m in 2100 (1.88m), within a 90% C.I. of -0.03-5.97m. Thus, the latter projections are much higher with much greater uncertainty. Our method of constructing a probability distribution using the ABUMIP dataset means that the range of our SLR distribution corresponds exactly to the difference between the lowest and highest models in ABUMIP, with the median towards the lower end of the range since most models are clustered there.

Figure SI9. Projection of Antarctica's SLR contribution this century from [16] (top) and [17] (bottom). Projections are relative to 2000. White line represents median value; dark shaded area represents the 67% confidence interval; light shaded area represents the 90% confidence interval. Results from a Monte Carlo simulation with sample size 50,000.

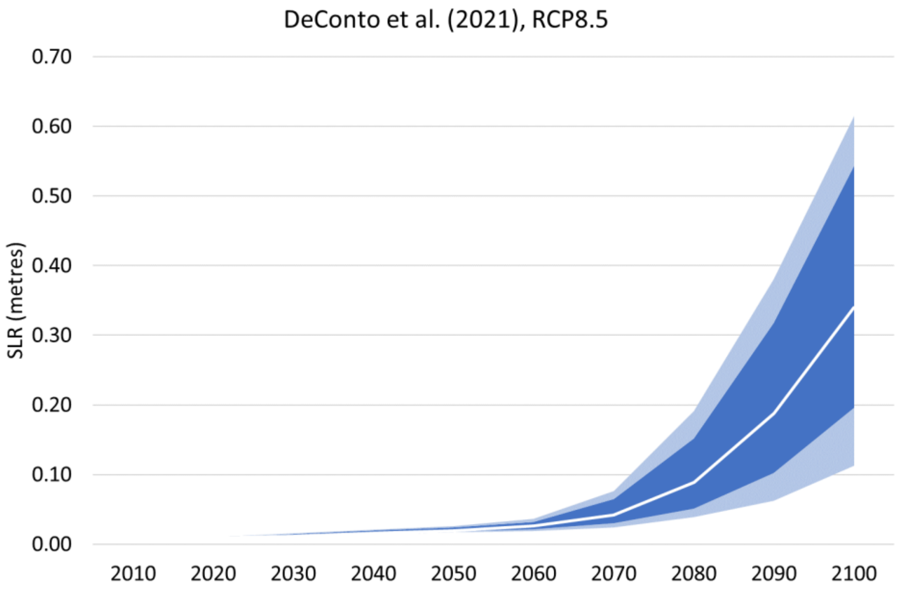

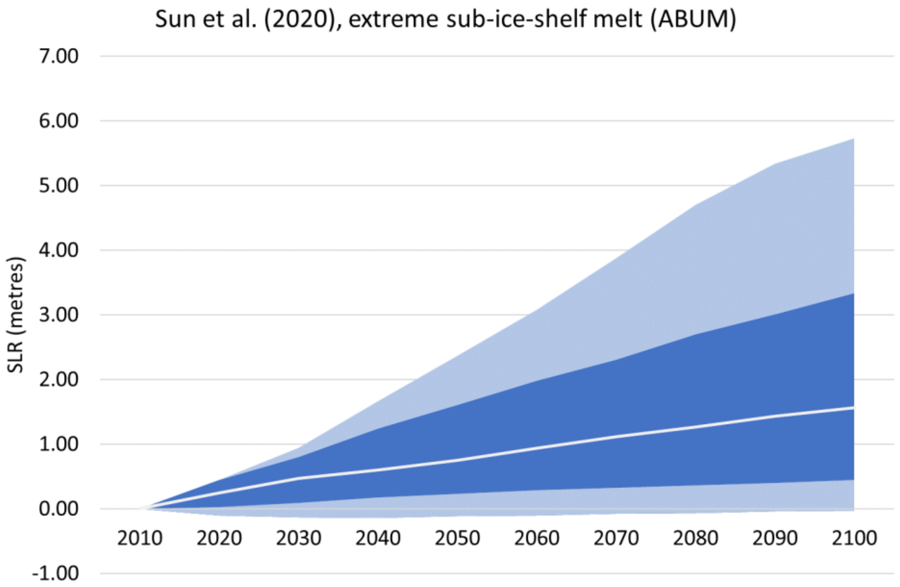

Figure SI10 compares the discounted NPV of SLR costs on different scenarios, taking a longer-term perspective than Figure 5 of the main paper; 2200 compared with 2100. Costs are higher on higher emissions scenarios. They are also higher on RCP8.5 using the projections of [16] instead of our main projections based on the LARMIP-2 models [3]. This is consistent with the fact that the DeConto et al. SLR projections are notably high only after 2100 (see [16] Figure 1(h)). NPV costs are much higher when using the extreme ice shelf melting scenario results of the ABUMIP project [17].

Figure SI10. Discounted net present value (in 2020 US dollars using 4% discount rate) of global adaptation and residual damage costs up to 2200, assuming no adaptation (left) and least-cost adaptation (right), for different scenarios. Error bars show the 90% confidence interval arising from global mean SLR uncertainty due to AIS melting.

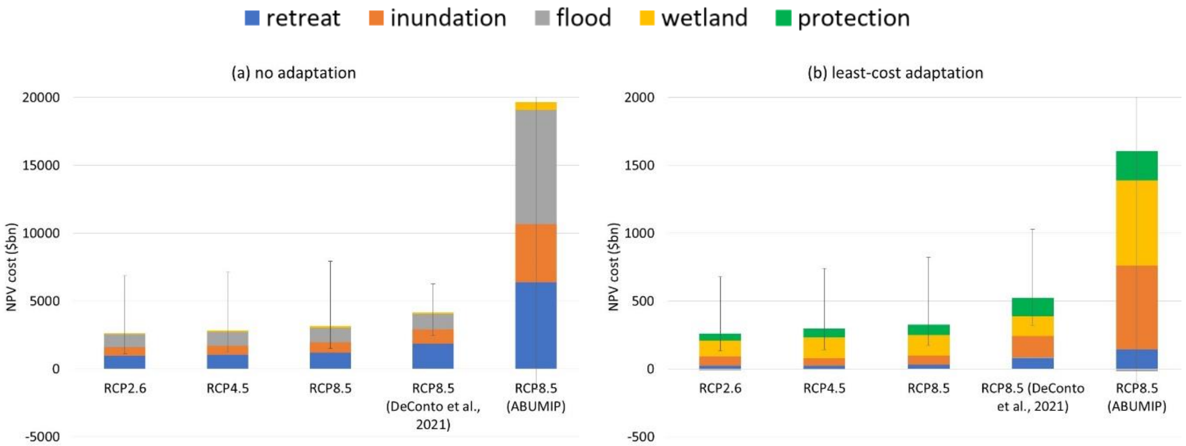

Figure SI11 shows the distribution of costs from AIS melting across countries at the end of the century and accompanies the analysis in Figure 4 of the main paper, which looks at the distribution mid-century instead. Again, the distribution of costs is highly unequal, with a small 'tail' of countries experiencing high costs relative to the projected size of their national economies. Many of these highly exposed countries are SIDS but compared with the mid-century projections the group is more diverse. Assuming no protection, several countries in the Middle East and African region are in the top ten. Assuming least-cost adaptation, Australia faces notably high costs relative to GDP, as its assets/population are mostly located by the coast, and GDP grows more slowly than in lower-income countries, thus absolute costs matter more.

Figure SI11. Country-level costs as a percentage of GDP at the end of the 21st century (2090-2110 average) on RCP4.5. Top panel shows no protection scenario, bottom panel shows least-cost adaptation scenario.

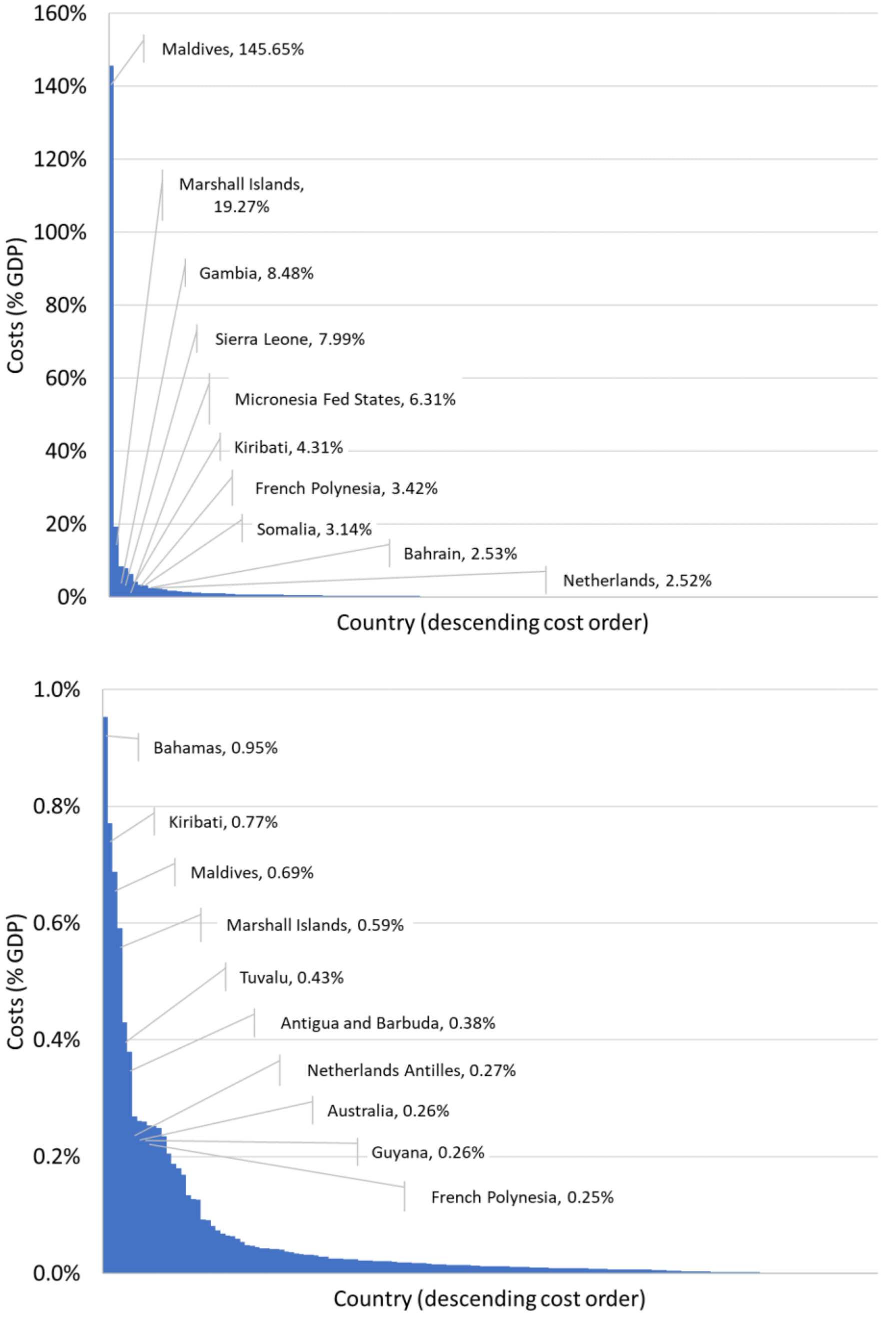

Social cost of carbon under alternative discount rates

Figure SI12 shows the contribution of SLR from AIS melting to the social cost of carbon under alternative assumptions about the pure rate of time preference.

Figure SI12. Box and whisker plots showing the incremental contribution of SLR from AIS melting to the social cost of carbon given a pure rate of time preference of 0.1% (top panel) and 2% (bottom). Boxes show median and interquartile ranges, whiskers show 95% CI, crosses mark the average change (0.1% trimmed), triangles mark the 0.5 percentile, and squares mark the 99.5 percentile. Elasticity of marginal utility is 1.5; mixed levels/growth damages (phi=0.5). Results from Monte Carlo simulations with sample size 10,000.

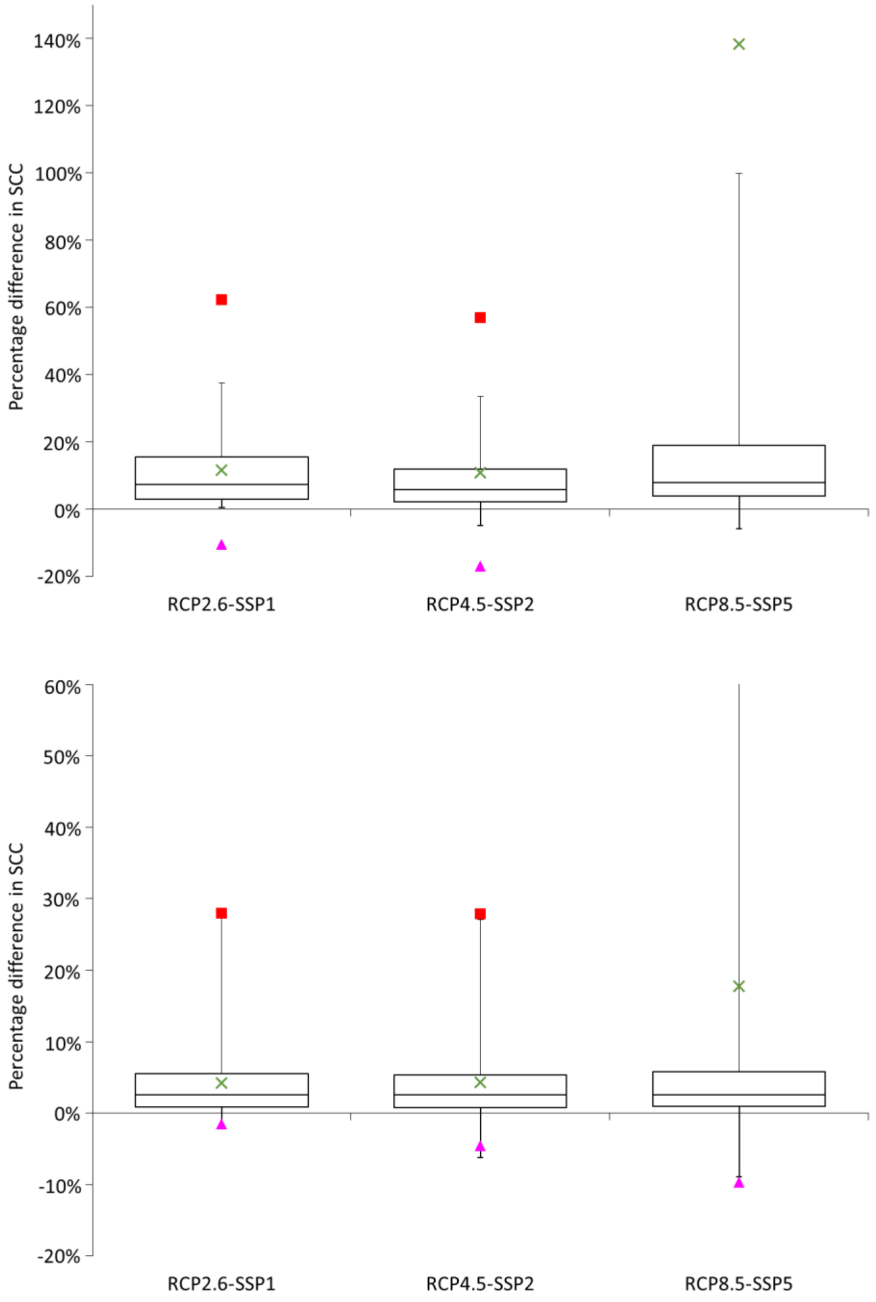

References

1. Frieler, K. *et al.* Consistent evidence of increasing Antarctic accumulation with warming. *Nat. Clim. Chang.* **5**, 348–352 (2015).
2. Garbe, J., Albrecht, T., Levermann, A., Donges, J. F. & Winkelmann, R. The hysteresis of the Antarctic ice sheet. *Nature* **585**, 538–544 (2020).
3. Levermann, A. *et al.* Projecting Antarctica's contribution to future sea level rise from basal ice shelf melt using linear response functions of 16 ice sheet models (LARMIP-2). *Earth Syst. Dyn.* **11**, 35–76 (2020).
4. Kopp, R. E. *et al.* Probabilistic 21st and 22nd century sea-level projections at a global network of tide-gauge sites. *Earth's Futur.* **2**, 383–406 (2014).
5. Diaz, D. B. Estimating global damages from sea level rise with the Coastal Impact and Adaptation Model (CIAM). *Clim. Change* **137**, 143–156 (2016).
6. Hinkel, J. & Klein, R. J. T. Integrating knowledge to assess coastal vulnerability to sea-level rise: The development of the DIVA tool. *Glob. Environ. Chang.* **19**, 384–395 (2009).
7. Dietz, S., Rising, J., Stoerk, T. & Wagner, G. Economic impacts of tipping points in the climate system. *Proc. Natl. Acad. Sci.* **118**, e2103081118 (2021).
8. Meinshausen, Malte, S. J. S. *et al.* The RCP greenhouse gas concentrations and their extensions from 1765 to 2300. *Clim. Change* **109**, 213–241 (2011).
9. Millar, R. J., Nicholls, Z. R., Friedlingstein, P. & Allen, M. R. A modified impulse-response representation of the global near-surface air temperature and atmospheric concentration response to carbon dioxide emissions. *Atmos. Chem. Phys.* **17**, 7213–7228 (2017).
10. Geoffroy, O. *et al.* Transient climate response in a two-layer energy-balance model. Part I: Analytical solution and parameter calibration using CMIP5 AOGCM experiments. *J. Clim.* **26**, 1841–1857 (2013).
11. Burke, M. B., Hsiang, S. M. & Miguel, E. Global non-linear effect of temperature on economic production. *Nature* **527**, 235–239 (2015).
12. Diaz, D. B. & Keller, K. A potential disintegration of the West Antarctic Ice Sheet: implications for economic analyses of climate policy. *Am. Econ. Rev. Pap. Proc.* **106**, 607–11 (2016).
13. Nordhaus, W. D. Economics of the disintegration of the Greenland ice sheet. *Proc. Natl. Acad. Sci.* **16**, 12261–12269 (2019).
14. Newell, R. G., Prest, B. C. & Sexton, S. E. The GDP-temperature relationship: implications for climate change damages. *J. Environ. Econ. Manage.* **108**, 102445 (2021).
15. Baylor Fox-Kemper *et al.* Ocean, Cryosphere and Sea Level Change. in *Climate Change 2021: The Physical Science Basis. Contribution of Working Group I to the Sixth Assessment Report of the Intergovernmental Panel on Climate Change* (eds. Masson-Delmotte, V. et al.) (Cambridge University Press, 2021).
16. DeConto, R. M. *et al.* The Paris Climate Agreement and future sea-level rise from

Antarctica. *Nature* **593**, 83–89 (2021).

17. Sun, S. *et al.* Antarctic ice sheet response to sudden and sustained ice-shelf collapse (ABUMIP). *J. Glaciol.* **66**, 891–904 (2020).

18. Bindschadler, R. A. *et al.* Ice-sheet model sensitivities to environmental forcing and their use in projecting future sea level (the SeaRISE project). *J. Glaciol.* **59**, 195–224 (2013).
